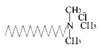# Green Preparation of Aqueous Graphene Dispersion and Study on Its Dispersion Stability

**DOI:** 10.3390/ma13184069

**Published:** 2020-09-14

**Authors:** Liangchuan Li, Ming Zhou, Long Jin, Youtang Mo, Enyong Xu, Huajin Chen, Lincong Liu, Mingyue Wang, Xin Chen, Hongwei Zhu

**Affiliations:** 1 School of Mechanical and Transportation Engineering, Guangxi University of Science and Technology, Liuzhou 545006, China; liliangc06@163.com (L.L.); 100001546@gxust.edu.cn (L.J); 100001794@gxust.edu.cn (Y.M.); 15177728580@163.com (L.L.); mingyuewang61@163.com (M.W.); max179356898@163.com (X.C.); 2 Dongfeng Liuzhou Motor Co., Ltd., Liuzhou 545006, China; 13557729070@163.com; 3 School of Electrical and Information Engineering, Guangxi University of Science and Technology, Liuzhou 545006, China; hjchen@gxust.edu.cn; 4 State Key Laboratory of New Ceramics and Fine Processing, School of Materials Science and Engineering, Tsinghua University, Beijing 100084, China; hongweizhu@tsinghua.edu.cn

**Keywords:** expanded graphite, liquid-phase exfoliation, aqueous graphene dispersion, graphene layers, defect density

## Abstract

The large-scale preparation of stable graphene aqueous dispersion has been a challenge in the theoretical research and industrial applications of graphene. This study determined the suitable exfoliation agent for overcoming the van der Waals force between the layers of expanded graphite sheets using the liquid-phase exfoliation method on the basis of surface energy theory to prepare a single layer of graphene. To evenly and stably disperse graphene in pure water, the dispersants were selected based on Hansen solubility parameters, namely, hydrophilicity, heterocyclic structure and easy combinative features. The graphene exfoliation grade and the dispersion stability, number of layers and defect density in the dispersion were analysed under Tyndall phenomenon using volume sedimentation method, zeta potential analysis, scanning electron microscopy, Raman spectroscopy and atomic force microscopy characterization. Subsequently, the long-chain quaternary ammonium salt cationic surfactant octadecyltrimethylammonium chloride (0.3 wt.%) was electrolyzed in pure water to form ammonium ions, which promoted hydrogen bonding in the remaining oxygen-containing groups on the surface of the stripped graphene. Forming the electrostatic steric hindrance effect to achieve the stable dispersion of graphene in water can exfoliate a minimum of eight layers of graphene nanosheets; the average number of layers was less than 14. The 0.1 wt.% (sodium dodecylbenzene sulfonate: melamine = 1:1) mixed system forms π–π interaction and hydrogen bonding with graphene in pure water, which allow the stable dispersion of graphene for 22 days without sedimentation. The findings can be beneficial for the large-scale preparation of waterborne graphene in industrial applications.

## 1. Introduction

Graphene^1^ is a 2D nanomaterial composed of carbon atoms that are compactly formed through the hybridization of $sp^{2}$ and has the thickness of one carbon atom (0.334 nm) [1,2]. This material possesses excellent electrical [3], mechanical [4], thermal [5] and optical properties [6], and has become the key research object in the new generation of 2D nanomaterials. Graphene is widely used in the fields of energy storage [7], electronic devices [8], photocatalysis [9], composite materials [10], sensing [11], biomedicine [12] and national defense [13]. The large-scale preparation of high-quality graphene is the only way to commercialize the experimental research of this field [14]. The preparation of graphene can be performed using physical, chemical, electrochemical exfoliation, and other methods [15]. The most classic chemical preparation method is the vapor deposition method [16], which can obtain high-quality single-layer graphene. However, chemical methods have a long preparation cycle, high energy consumption, complicated process and harsh preparation conditions [4]. Conversely, physical methods support green economy, have a short preparation cycle and simple process and produces few defects [17].

At present, the liquid-phase exfoliation method poses the highest potential for the large-scale preparation of graphene amongst the physical preparation methods [18]. This method aims to select the exfoliation agent that matches the surface energy of graphene, according to surface energy theory [19]. In addition, this approach obtains and stably disperses a few layers (or single-layer) [20] in the liquid medium to overcome the van der Waals force between the layers of the graphene sheet. To prevent the agglomeration behavior of graphene [21], an appropriate dispersant–solvent system was developed on the basis of the Hansen solubility parameters (HPSs) [22], that was, a dispersant that was hydrophilic, heterocyclic, and easy to combine with graphene was used to physically adsorb (e.g., electrostatic [23], hydrogen bonding [21] and π–π interactions [24]) were combined on the surface of graphene to prevent the agglomeration of adjacent graphene nanoplatelets and make them stably dispersed in various types of liquid media. Moreover, the surfactants [25], ionic liquids [26], amidine derivatives [27] and proteins [28] must have exfoliation and dispersing ability. Many researchers have applied liquid-phase exfoliation theory to study a surfactant-free graphene dispersion system. However, despite achieving biosafety and satisfying the requirements of green economic production, the concentration of graphene in the dispersion prepared using such approach was lower than that in the surfactant dispersion system, and the quality (e.g., number of layers and defect density) of the former was inferior to that of the latter. Wang et al. [29] prepared graphene dispersion by ultrasonically exfoliating graphite in an aqueous solution containing Li^+^ and OH^−^. During the process, Li^+^ entered the layers of the graphite sheets to promote the exfoliation of graphene. OH^−^ was then added to the surface of the graphene to increase its hydrophilicity and dispersion stability in aqueous media. This dispersion theory did not include any surfactants and the entire experimental process was simple, low-cost and environmental-friendly. However, the stable dispersion of the surfactant-free system of graphene in the dispersion was far from that of the surfactant system, and it does not have the potential for the large-scale preparation of graphene [30]. Therefore, the large-scale and rapid preparation of high-concentration and high-quality graphene dispersions is still dominated by surfactant dispersion systems. Many research groups have used two dispersants to study the preparation of graphene dispersions. During the preparation of graphene dispersions, Mahdiyeh et al. [31] discovered that the dispersion of the mixture of pure sodium dodecyl sulphate (SDS) and anionic surfactants in aqueous media was greater than that of pure hexadecyl trimethyl ammonium bromide (CTAB) and cationic mixtures. This result may be because the short chain tail and small particle head of SDS avoid electrostatic repulsion with the same SDS tail and head–head, and can be more adsorbed on the surface of graphene and exhibit a cooperative dispersion effect with anionic surfactants. Additionally, Sham et al. [32] studied the stable dispersibility of graphene in various dispersants in aqueous media and found that Na^+^ salt surfactants have the best wettability for graphene, which can promote the aqueous dispersion of graphene. Therefore, Na^+^-containing anions and other types of surfactants were selected as dispersants (see the Table 1).

In this study, the liquid-phase exfoliation method was used to prepare a graphene dispersion. On the basis of surface energy theory and HPSs, the exfoliation agent that will be used as the dispersant must have a surface energy that is close to that of graphene, hydrophilic property and heterocyclic structure and should be easy to combine with graphene. The dispersants that fit these criteria are listed in Table 1. Green economical ultrasonic degradation process was performed in aqueous media to exfoliate the expanded graphite and prepare the graphene dispersion. The surface agent with different mass fractions and the exfoliation and dispersion stability of graphene were also studied. Unlike the method of Mahdiyeh et al. [31], this study mixed melamine, a small molecule heterocyclic non-ionic surfactant, with ionic surfactant sodium dodecylbenzene sulfonate (SDBS) and non-ionic surfactant polyvinyl pyrrolidone (PVP) to investigate the exfoliation ability and stable dispersion of graphene. In aqueous solution, the volumetric sedimentation method and the Tyndall phenomenon analyzed the exfoliation grade and dispersion ability of graphene dispersant system. The dispersion ability of each dispersant system and the stable dispersion mechanism were analysed through scanning electron microscopy, Raman spectroscopy (Raman) and atomic force microscopy characterization, and the number of layers and defect density of graphene in the dispersion were discussed.

## 2. Experiment

### 2.1. Reagents and Equipment

The expanded graphite (carbon content: 95%) was purchased from Tsinglube Technology Co., Ltd. (Liuzhou, China), the ionic liquid was obtained from Lanzhou Institute of Chemical Physics (Lanzhou, China), Chinese Academy of Sciences and the other analytical grade reagents were purchased from Shanghai Aladdin Bio-Chem Technology Co., Ltd. (Shanghai, China).

The main instruments used in the experiment include: scanning electron microscope (SEM), Carl Zeiss (Cambridge, Germany); atomic force microscope (AFM, Probe Model: HQ-300-Au, Lot#: 180810), Oxford Instruments (Shanghai, China); Raman, laser model: 532nm, XPOLORA PLUS, JY-HR800 (HOBIBA, Tokyo, Japan); Zetasizer Nano ZS (Malvern, London, Britain).

### 2.2. Experiment Section

**Figure 1:**
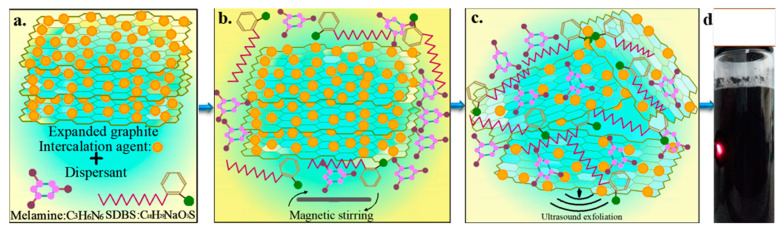
Schematic of the preparation of graphene dispersion: (**a**) expanded graphite and dispersant mixed system, (**b**) magnetically stirred expanded graphite and uniformly mixed dispersant system, (**c**) ultrasonic peeling of expanded graphite to prepare the graphene dispersion and (**d**) ultrasonic exfoliation and dispersion of graphene.

The expanded graphite, which was dried at 80 ± 0.5 °C for 24 h, and a dispersant were added to pure water and magnetically stirred at 60 ± 0.5 °C for 1 h. Subsequently, ultrasonic exfoliation was performed at 60 ± 2.5 °C for 8 h. The mass fraction of the expanded graphite accounts for 0.5 wt.% of the total system mass and that of the dispersant varies at 0.1, 0.3, 0.5 and 1 wt.% of the total system. Except for the NaOH dispersion system, the pH of all dispersant systems was 7.1 ± 0.15. The microscopic mechanism of the 0.5 wt. % dispersant system (SDBS: melamine = 1:1), which was used as a trial basis for the 0.1, 0.3 and 1 wt.% dispersant systems, is shown in Figure 1.

## 3. Results and Discussion

### 3.1. Analysis of the Exfoliation Grade and Dispersion Stability

**Figure 2:**
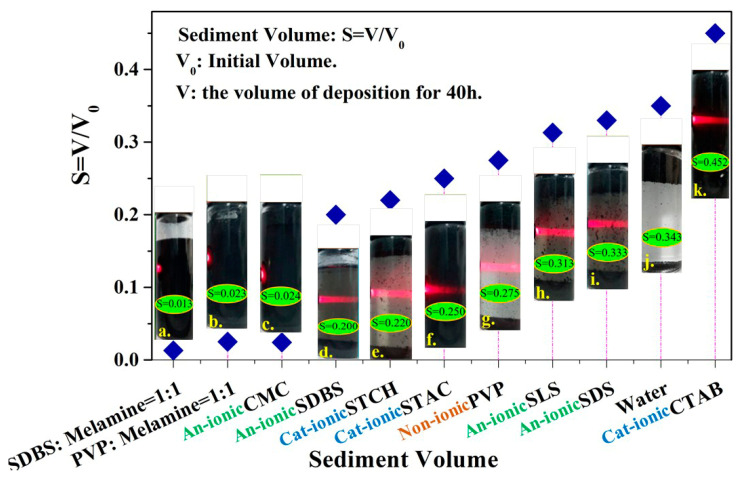
Tyndall effect and volume sedimentation test results of a 0.5 wt.% dispersant system.

The exfoliation grade determines the quality and application value of graphene (e.g., number of layers, defect density, lateral dimensions and stable dispersion) in liquid media. The Tyndall phenomenon is a common method for estimating the particle size of the solution in the liquid-phase system after exfoliation. This method relies on the size of the scattering effect of light when parallel light passes through the dispersed phase to evaluate the exfoliation size of graphene in the dispersion. The 0.5 wt.% dispersant system was tested by irradiating parallel light in Figure 2. The 0.5 wt.% CTAB, octadearyl dimethyl ammonium chloride (STAC), SDS, sodium lauryl sulfonate (SLS) and SDBS systems demonstrated remarkable Tyndall phenomena, which suggests that the particle diameter of the graphene after the exfoliation in the dispersion ranges from 1–100 nm. The degree of scattering of the parallel light passing through the dispersed phase and the exfoliation ability and dispersion effect of the quaternary ammonium salt cationic surfactants on graphene were better than those of other ionic and non-ionic surfactants in this experiment. Moreover, the mixed system of cyclic anionic surfactant and heterocyclic non-ionic surfactant displayed a more stable dispersibility for graphene than the single dispersant system.

The volume sedimentation method is one of the simplest and most convenient methods for detecting the stable dispersion of colloids. The smaller the volume ratio of the sediment in the dispersed phase, the higher the concentration of the graphene in the dispersion. The dispersion solution volume ratio is determined as $S=V/V_{0}$, where V is the volume value of the sediment in the dispersed phase after the sample is deposited for 40 h and $V_{0}$ is the total volume value of the dispersed phase during the initial deposit of the sample. The dispersion of graphene in the solution was more effective when the value of S is small. Figure 2 shows the S value of the graphene dispersion in the different 0.5 wt.% dispersant systems after depositing the sample for 40 h. The results show that the SDBS (SDBS: melamine = 1:1 and PVP: melamine = 1:1) and carboxymethylcellulose sodium (CMC) systems exhibit the most satisfactory dispersion stability (*S* value ≤ 0.03). These findings are consistent with the findings of Majid et al. [33] and Mateos et al. [34], who reported that ionic dispersants provided stronger promotion of the stable graphene dispersion ability of each surfactant in pure water compared with non-ionic ones. In addition, the (SDBS: melamine = 1:1) graphene can be stably dispersed for 22 days without sedimentation. This result has a crucial implication for industrial production. For instance, the stable dispersibility of graphene is an extremely important indicator in the application of water-based graphene-modified cement concrete.

**Figure 3:**
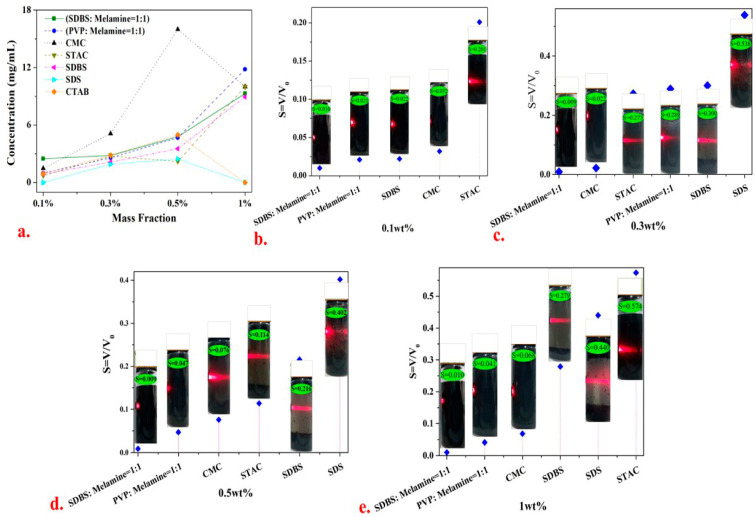
Volume settling experiment using a dispersion system with dispersant mass fractions of 0.1, 0.3, 0.5 and 1 wt.%: (**a**) statistical results of solute concentration and (**b–e**) volume settlement results of each dispersion system after 40 h.

The mass fractions of (SDBS: melamine = 1:1) (PVP: melamine = 1:1) CMC, STAC, SLS and SDBS systems (0.1 wt.%, 0.3 wt.% and 1 wt.%) were varied to further analyzed the graphene dispersion stability in Figure 3. The results show that as the mass fraction of the dispersant in the dispersion system increases from 0.1 wt.% to 1.0 wt.%, the exfoliation grade and dispersion stability of graphene deteriorate, which is in agreement with the results obtained by Nawaz et al. [35] in their dispersion experiment involving aqueous graphene. The dispersion stability (*S* < 0.05) of the mixed dispersant system (SDBS: melamine = 1:1) was superior to those of single dispersant systems, indicating that the synergistic effect of SDBS and melamine improves the dispersion stability of graphene in pure water. The most excellent dispersion stability was observed when the mass fraction of STAC and CMC in the single dispersant system was 0.3 wt.%. If the mass fraction of the dispersant is excessively high, the dispersant exhibits a micelle phenomenon and loses the ability to stabilize the dispersion. If the mass fraction is extremely low, the dispersion effect cannot be achieved. The solute concentration in each dispersant system was statistically measured in Figure 3a. The entire CMC system has the highest solute concentration amongst the systems and the 0.5 wt.% CMC system displayed the highest concentration. 

Given the existence of the critical micelle concentrations of the surfactants (CMC, CTAB and SDS), the best graphene dispersion effect appears near the micelle concentration. The mass fraction of the dispersants was extremely high due to the intermolecular similarity. The interaction force [35] shows that the self-phase stacking phenomenon loses the graphene dispersion stability; if such force is extremely low, no stable dispersion effect will manifest on graphene.

**Figure 4:**
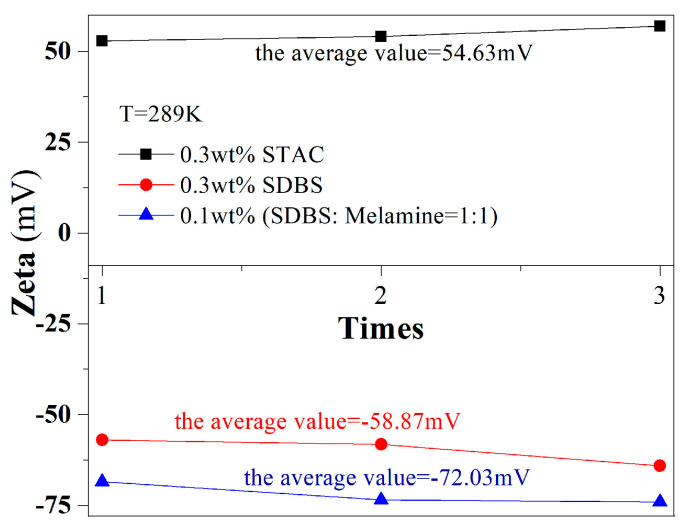
Zeta potential values of the 0.3 wt.% STAC, 0.3 wt.% SDBS and 0.1 wt.% (SDBS: melamine = 1:1) systems.

After the analysis, the chain cationic surfactant STAC was easily electrolyzed to form ammonium ions in the water, which promoted hydrogen bonding with the remaining oxygen-containing groups on the graphene surface after exfoliation [21]. This bonding formed an electrostatic potential resistance that is greater than the van der Waals force between graphene sheets [36] and promoted the stable dispersion of graphene in water. The average zeta potential of the dispersion system was 54.63 mV (>30 mV in Figure 4). However, CTAB, which was a dispersant with similar physical and chemical properties, only dissolves in warm water and thus caused severe precipitation phenomena at room temperature in Figure 2k. In conclusion, CTAB was not an ideal dispersant for experimental research and industrial applications.

**Figure 5:**
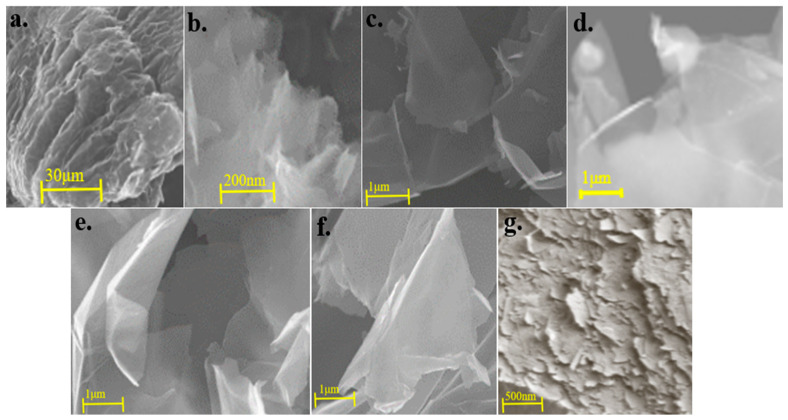
(**a**). SEM images of expanded graphite; (**b**). SEM images of 0.1 wt.% system (surfactant sodium dodecylbenzene sulfonate (SDBS): melamine = 1:1) graphene; (**c**). SEM images of 0.3 wt.% octadearyl dimethyl ammonium chloride (STAC) dispersion system graphene; (**d**). SEM images of 0.3 wt.% carboxymethylcellulose sodium (CMC) system graphene; (**e**). SEM images of 0.1 wt.% SDBS system graphene; (**f**). SEM images of 0.3 wt.% SDBS systems graphene; (**g**). SEM images of graphene prepared by Phiri et al.

The dispersion stabilities of 0.1 wt.% and 0.3 wt.% SDBS and 0.3 wt.% CMC systems were evident in Figure 3b,c. As shown in Figure 5d–f, after the SEM characterization, a transparent single-layer graphene appeared, a large number of dispersants were adsorbed between the graphene sheets and the proportion of the few graphene layers is high. Given that CMC and SDBS were extremely hydrophilic and contain heterocyclic structures, steric hindrance can be generated between the graphene sheet layers, thereby preventing the graphene agglomeration behavior.

In non-ionic surfactant systems, although PVP can significantly reduce the surface tension of aqueous dispersion systems, the graphene nanosheets in the water are only affected by the steric hindrance of PVP, which was smaller than the interlayers of the graphene sheets. The van der Waals force cannot promote the stable dispersion of graphene in water. The average zeta potential value was −72.03 mV (absolute value >30 mV, in Figure 4), which proves that the dispersion stability of graphene in the 0.1 wt.% (SDBS: melamine = 1:1) system was significant.

Phiri et al. [37] used a high-shear exfoliation mixer to facilitate the ultrasonic exfoliation of graphite and prepare a graphene dispersion in pure water. The shear force generated by the high-speed shearing peeler was used to overcome the van der Waals force between the graphene sheets to increase the yield of graphene during ultrasonic exfoliation. As shown in Figure 5g, the quality of the graphene (e.g., layer number, defect density and lateral size) in the systems prepared in this study was superior to that of the graphene prepared by Phiri et al. The graphene dispersion process was an important issue in the large-scale preparation of high-quality graphene dispersions but not the core issue in the research on graphene dispersion. However, graphene dispersant theory was the primary focus of the research on the liquid-phase dispersion of graphene.

### 3.2. Graphene Layers

**Figure 6:**
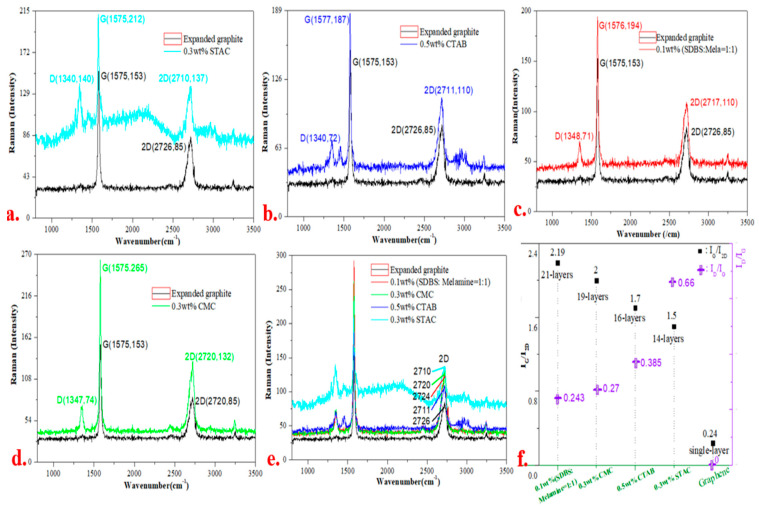
Raman diagram of graphene: (**a–d**) Raman diagram of graphene in the 0.3 wt.% STAC, 0.5 wt.% pure hexadecyl trimethyl ammonium bromide (CTAB), 0.1 wt.% (SDBS: melamine = 1:1) and 0.3 wt.% CMC systems, respectively; (**e**) comparison of the Raman characteristic peak intensities of graphene and expanded graphite; and (**f**) calculation results of the Raman characteristic peaks (I_G_/I_2D_ and I_D_/I_G_) and number of graphene layers for each dispersed system.

The most common methods for characterizing graphene layers include Raman and atomic force microscopy; the graphene in each dispersed system was characterized using the former (Figure 6). Compared with the characteristic peaks of the expanded graphite in Figure 6a–d, the 0.3 wt.% STAC, 0.5 wt.% CTAB, 0.1 wt.% (SDBS: melamine = 1:1) and 0.3 wt.% CMC systems significantly enhanced the graphene characteristic peak signals. In addition, the 2D peak positions of graphene in each dispersant system show red shift phenomenon in Figure 6e [38]. The number of layers of the expanded graphite in each dispersant system has been exfoliated in varying degrees.

On the basis of the value of I_G_ / I_2D_ in the Raman characteristic peaks of graphene [39], the average number of graphene layers in each dispersant system was 14–21 layers in Figure 6f; the average number of graphene layers in the 0.3 wt.% STAC is the lowest amongst the systems (approximately 14 layers).

**Figure 7:**
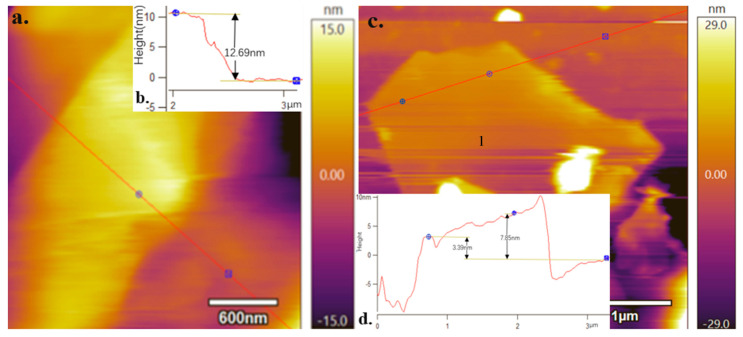
(**a**) Atomic force microscopy (AFM) diagram of graphene in the 0.1 wt.% (SDBS: melamine = 1:1) system; (**b**) surface height graph of (a); (**c**) AFM diagram of graphene in the 0.3 wt.% STAC system (‘1’ represents the protruding point of the graphene edge folds); (**d**) surface height curve of (**c**).

The graphene in each dispersant system was also characterised through AFM. A distinct graph of the graphene layer structure was observed in the 0.1 wt.% (SDBS: melamine = 1:1) and 0.3 wt.% STAC systems in Figure 7a,c. The surface height of the graphene was then calibrated as follows: |dz|_1_ = 12.69 nm, |dz|_2_ = 7.85 and 3.39 nm (as seen Figure 7b,d). Wen et al. [40] exfoliated and dispersed the expanded graphite to prepare graphene and revealed that the interlayer distance of the expanded graphite was 0.43 nm. Subsequently, the number of graphene layers in the 0.1 wt.% (SDBS: melamine = 1:1) system was calculated on the basis of the interlayer distance. The obtained number of layers for this system was at most 29, whereas that in the 0.3 wt.% STAC dispersant system was at most eight.

The results of the Raman and AFM analyses indicate that the average numbers of graphene nanoflakes in the 0.1 wt.% (SDBS: melamine = 1:1) and 0.3 wt.% STAC systems are approximately 14 and 21, respectively, and a minimum of eight layers of graphene nanoflakes can be exfoliated out.

### 3.3. Defect Density of Graphene

Figure 6 illustrates D peaks, which indicate that each dispersed system contains defects to varying degrees in comparison with the sp^2^ hybrid-structured intact expanded graphite. The defect density of graphene in the 0.3 wt.% STAC system is the largest amongst all systems in the experiment (I_D_/I_G_ = 0.66). The defects, which are generated in the ultrasonic exfoliation process, produce wrinkles. For instance, the point designated by ‘1’ in Figure 7d was caused by the wrinkles generated at the edges of the graphene. Graphene contains few defects, which can improve the dispersibility of graphene in various types of nanocomposites. In addition, graphene nanosheets with controllable defect density have a certain number of band gaps on the surface, which has great application value for graphene in electronics, sensing and other fields.

## 4. Conclusions

This study explored the use of a suitable stripping agent in pure water, based on surface energy theory, to overcome the van der Waals force between graphene sheets and prepare graphene. The exfoliation agents must be hydrophilic, heterocyclic and easy to combine with graphene. The graphene surface interacts with a dispersant to prepare few layers of uniformly dispersed aqueous graphene dispersion. The following conclusions were obtained after the experiments:

(1) The electrostatic repulsion force of the charged particles formed by the ionisation of the quaternary ammonium cationic surfactants in water was greater than the steric resistance generated by the non-ionic surfactants. The former also exerts a strong effect on the van der Waals force between the graphene sheets and enhances the exfoliation ability and dispersion stability. The 0.3 wt.% STAC dispersant system exhibited excellent exfoliation ability and dispersion stability and can exfoliate graphene nanosheets with less than eight layers (the average number of layers was less than 14 layers). In addition, the graphene can be stably dispersed in water and no settlement occurred for 13 days.
(2) The mixed system of cyclic anionic surfactants and heterocyclic non-ionic surfactants exerts a complex dispersion effect on graphene through electrostatic repulsion and π–π action, which was more stable than that of a single dispersant system. The graphene in the 0.1 wt.% (SDBS: melamine = 1:1) system can remain stable in the storage for 22 days without sedimentation.

This study used green dispersant to prepare a few layers of stable graphene dispersion in an aqueous system, which is of great significance for the large-scale preparation of such dispersions in practical industrial applications.

## Figures and Tables

**Table 1:** Summary of the molecular formula, structural formula and type of various dispersants.

Dispersant	Molecular Formula	Structural Formula	Category
Sodium hydroxide	NaOH		Strong base
1-Butylpyridinium bis((trifluoromethyl)sulfonyl)imide	C_11_H_14_F_6_N_2_O_4_S_2_	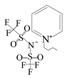	Ionic liquid
1-Butylpyridinium tetrafluoroborate	C_9_H_14_BF_4_N	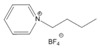	
Melamine	C_3_H_6_N_6_	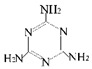	Non-ionic surfactant
Triton X-100	C_34_H_62_O_11_	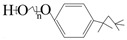	
PVP	(C_6_H_9_NO)n	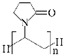	
Sodium pyrophosphate	Na_4_P_2_O_7_	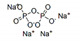	Dispersant
Sodium citrate (anhydrous)	C_6_H_5_Na_3_O_7_	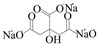	
Sodium taurodeoxycholate hydrate	C_26_H_44_NNaO_6_S	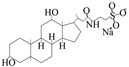	Anionic surfactant
Sodium lauryl sulfonate (SLS)	C_12_H_25_SO_3_Na	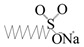	
SDS	C_12_H_25_SO_4_Na	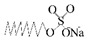	
SDBS	C_18_H_29_NaO_3_S	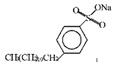	
Carboxymethylcellulose sodium (CMC)	C_8_H_16_NaO_8_	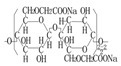	
CTAB	C_19_H_42_NBr	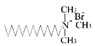	Cationic surfactant
Octadearyl dimethyl ammonium chloride (STAC)	C_21_H_46_NCl